# Examining the Effects of Viewing Nature and Animal Smartphone Wallpapers on Affect, Behaviour, and Cognition: A Randomised Cross-Over Trial

**DOI:** 10.3390/bs16050623

**Published:** 2026-04-22

**Authors:** Nadyanna M. Majeed, Nicole R. Y. Chen, Adalia Y. H. Goh, Meilan Hu, Kenneth J. J. Koh, Yuolmae H. G. Ang, Andree Hartanto

**Affiliations:** 1 Department of Psychology, Faculty of Arts and Social Sciences, National University of Singapore, Singapore 117570, Singapore; 2 School of Social Sciences, Singapore Management University, 10 Canning Rise, Singapore 179873, Singapore; 3 School of Art, Design and Media, Nanyang Technological University, Singapore 639798, Singapore

**Keywords:** animal, nature, well-being, lock screen, wallpaper, smartphone

## Abstract

This study aims to investigate the effects of different smartphone lock screen wallpapers on weekly perceived well-being, procrastination, and productivity in young adults. Through a pre-registered within-subject experiment, 60 participants were exposed to three smartphone wallpaper conditions: nature, animal, and neutral (control). Each participant experienced each condition over three weeks, with the order of conditions counterbalanced. Using Frequentist and Bayesian analyses, we did not find any differences between conditions across the pre-registered confirmatory outcomes (i.e., life satisfaction, positive affect, negative affect, stress, productivity, and procrastination). Exploratory outcomes related to lock screen engagement, however, revealed some meaningful effects. Animal-themed smartphone wallpapers increased recognition and distractibility, while nature-themed smartphone wallpapers were rated as more pleasing than both animal and control images. Our findings suggest that brief visual exposure to nature or animal-themed lock screens may be insufficient to influence broader well-being or behaviour. Implications for designing strategies to promote psychological health in everyday technology use are discussed.

## 1. Introduction

The psychological and physiological benefits of exposure to nature and animals have been well-documented. Natural environments are associated with reduced stress, improved mood, and enhanced overall well-being (Goh et al., 2023; Houlden et al., 2018; Yao et al., 2021; for recent meta-analysis, see Zeng et al., 2025). Likewise, exposure to animals—whether encountered in real life or viewed in images—has been shown to evoke feelings of comfort, reduce anxiety, and promote emotional regulation (Beetz et al., 2012; Correale et al., 2022; Yoo et al., 2024). These positive outcomes have often been interpreted through frameworks such as the Stress Reduction Theory (Ulrich et al., 1991) and the Biophilia Hypothesis (Wilson, 1984), both of which propose that humans are predisposed to respond positively to natural environments and animals (Kellert, 1997; Mormann et al., 2011). This connection can satisfy our psychological needs and enhance well-being (Howell & Passmore, 2013). Taken together, both nature and animals have the potential to serve as simple, yet powerful, interventions for improving well-being.

While nature and animals are often experienced together, they represent distinct entities that can independently contribute to well-being (Goh et al., 2023; Sheets & Manzer, 1991; Souter & Miller, 2007). Nature typically encompasses elements such as trees, mountains, water bodies, and plants (McMahan & Estes, 2015; Weeland et al., 2019). On the other hand, animals are living creatures that we interact with in both domestic settings and natural environments (Beetz et al., 2012; Chen et al., 2024). Interacting with nature, even in the absence of animals, has been shown to enhance well-being by reducing stress and improving mood (Fuegen & Breitenbecher, 2018; Passmore & Holder, 2017; South et al., 2018; Van Den Berg & Custers, 2011). A plethora of studies exist regarding human–nature interactions, spanning a range of designs, involving exposure times ranging from 10 min to 210 min (Yao et al., 2021), repeated multiple times in a day (Ojala et al., 2019; B.-J. Park et al., 2009) or across several days (Grazuleviciene et al., 2016), with activities varying from walking (A. M. Brooks et al., 2017; Elsadek et al., 2019) to viewing (Bielinis et al., 2018; J. Lee et al., 2009) and sitting in nature (J. Lee et al., 2011; B.-J. Park et al., 2010). Similarly, interactions with animals, whether in man-made environments like therapy sessions or homes, have demonstrated positive effects on physical and mental health (Barker & Wolen, 2008; Friedmann & Son, 2009; Le Roux & Kemp, 2009), as well as on interpersonal outcomes such as empathy and altruism (for review, see Chen et al., 2024). Furthermore, recent studies on online pet streaming have also highlighted the positive impact of animal imagery on subjective well-being (Zhou et al., 2020). Importantly, however, much of the literature has focused on live, interactive, or therapeutic contact with animals; comparatively less is known about whether brief, passive, and digitally mediated viewing of animals in everyday life yields similar benefits. While often intertwined, nature and animals uniquely and independently contribute to well-being in complementary ways.

While direct interaction with nature or animals provides the most tangible benefits, such experiences are not always accessible due to urbanisation, time constraints, and other practical barriers (Pedretti & Soren, 2006; Tan & Su, 2022). In response, researchers have examined whether digital representations of nature or animals can confer similar psychological benefits. Existing evidence suggests that such exposure can reduce negative affect and in some cases, promote positive affect (Table 1). However, this evidence base is dominated by studies using highly immersive modalities, particularly virtual reality (VR). Although effective, VR-based approaches rely on specialised and often costly equipment, limiting their feasibility for widespread, everyday use. Some studies have employed less immersive formats such as videos (Table 1), but these do not fully address the question of whether minimal, incidental exposure—integrated into routine daily interactions with personal devices—can yield comparable benefits. This gap underscores the need to examine more accessible and ecologically valid modalities, such as lock screen images.

In contrast, smartphones offer a practical and widely available platform for delivering low-intensity, repeated visual interventions in everyday life. From the perspective of Stress Reduction Theory, even brief exposure to natural cues can reduce stress (Haluza et al., 2025; Owens & Bunce, 2023). Likewise, the Biophilia Hypothesis suggests that visual representations of natural environments and animals may activate positively valenced associations, even when the stimuli are digitally mediated rather than directly experienced (Menzel & Reese, 2021). Smartphone lock screens may therefore serve as a low-cost, accessible, and scalable intervention vehicle. Unlike VR exposure, lock screen wallpapers do not require additional devices or active participation. Furthermore, the lock screen is repeatedly encountered throughout the day and is often the first visual stimulus seen during smartphone use (Diaz, 2022). Accordingly, lock screen wallpapers may offer a non-invasive, low-effort means of introducing potentially beneficial nature and animal imagery into daily life, leveraging the frequent and habitual interactions people have with their devices throughout the day (Heitmayer & Lahlou, 2021).

Despite the potential of a lock screen approach, no research to date has investigated whether such passive exposure via smartphone lock screen wallpapers could offer similar benefits. This is an important gap, as lock screen wallpapers differ in meaningful ways from the exposure forms more commonly examined in prior research: wallpapers are static, passive, brief but repeatedly encountered, and embedded within everyday smartphone use rather than delivered in therapeutic or laboratory-based sessions. The current work addresses this gap by investigating whether photos of nature and animals through smartphone lock screen wallpapers can improve everyday life outcomes (i.e., levels of stress, negative affect, positive affect, life satisfaction, procrastination, and productivity) through a pre-registered experiment. We distinguish our pre-registered outcome variables into primary psychological outcomes (i.e., stress, negative affect, positive affect, and life satisfaction) and secondary behavioural outcomes (i.e., procrastination and productivity). In line with Stress Reduction Theory (Ulrich, 1983) and the Biophilia Hypothesis (Wilson, 1984), we predict that exposure to lock screen wallpapers featuring calming natural scenes or animals will have the most immediate effects in reducing perceived stress and negative affect, and enhancing positive affect and life satisfaction. In turn, these primary psychological changes may support better day-to-day self-regulation, which could manifest secondarily in behaviours such as greater productivity and lower procrastination. Importantly, however, the present work is not designed to test a specific causal sequence among these outcomes. Rather, the work is consistent with a framework in which brief, repeated visual exposure may be associated first with psychological outcomes, and more broadly with everyday behavioural functioning.

Additionally, it is important to note that Stress Reduction Theory and the Biophilia Hypothesis were largely developed in relation to more immersive or sustained forms of exposure. In contrast, the present work focuses on brief and passive exposure to static digital images. Therefore, our aim is not to directly test these theories in their original scope, but to examine whether a highly minimal form of micro-exposure may still yield measurable benefits to well-being. Accordingly, we hypothesise that, relative to control wallpapers, nature and animal wallpapers would improve life satisfaction and positive affect, and reduce negative affect and perceived stress. We further hypothesise that these primary psychological outcomes would extend to downstream secondary behavioural outcomes, such that participants would report greater productivity and lower procrastination in the nature and animal conditions than in the control condition.

By examining the effects of these digital visual interventions, we aimed to explore the potential of simple, tech-assisted methods for promoting mental health in a world where direct interactions with nature and animals may be limited. To address this research question, we employed a pre-registered randomised cross-over trial design, where each participant experienced nature, animal, and control wallpaper conditions. This design was chosen to rule out between-person variability by allowing participants to serve as their own controls, thereby helping to account for stable individual differences. Including neutral wallpapers devoid of nature or animal imagery in the control condition further allowed us to ensure that any observed effects could be attributed to the specific nature or animal visuals, rather than the use of any generic visual. To ensure that the findings were robust, both Frequentist and Bayesian statistical approaches were incorporated.

## 2. Method

### 2.1. Sample and Power

All participants were recruited from a local university in Singapore in exchange for two course credits or SG$7.50 cash. Participants were required to be born from 1997 to 2007 inclusive to ensure the sample represented the Generation Z demographic (Dimock, 2019). Generation Z participants were specifically chosen for their distinct smartphone usage patterns and dramatic shifts in behaviours, attitudes, and lifestyles compared to those in older generations (Twenge & Campbell, 2018). As of 2019, 95% of Generation Z had access to a smartphone, with 45% reporting they are “almost constantly” online (Dimock, 2019). In addition, this age group is at a pivotal developmental stage where smartphone use can significantly impact well-being and productivity (Duke & Montag, 2017; Vahedi & Saiphoo, 2018). Participants were also required to own a smartphone compatible with the study (i.e., running either the iOS or Android system).

As pre-registered, we aimed for a sample size of 60. With *N* = 60, we would have 80% power to detect an effect size of η^2^*_g_* = 0.08 in a one-way repeated measures ANOVA with three levels (i.e., nature vs. animal vs. control conditions) at α = 0.05 based on Lakens and Caldwell’s (2021) *ANOVA exact* power tool.^1^

Participants were recruited from October 2023 to March 2025 inclusive. An initial sample of 60 individuals was recruited from late 2023 to early 2024. However, a technical error prevented 24 individuals from completing the final condition of the trial. Additionally, nine more responses were found to be incomplete. Therefore, an additional 33 individuals were recruited from late 2024 to early 2025 from the same source to ensure that the final sample consisted of exactly 60 participants (Table 2).^2^ Of this final sample, the vast majority (78%) were recruited in 2024.^3^

### 2.2. Design and Procedure

All 60 participants completed a baseline questionnaire, during which participants were briefed on the procedure of the experiment as well as the importance of adhering to the instructions and provided informed consent electronically. Participants were also informed that they would not be able to skip any questions, and that while they could withdraw from the study at any time without penalty, compensation would only be provided if the study was completed according to protocol. At all stages of data collection, the order in which measures were presented to participants was randomised to minimise order effects. In addition, the order of items within each measure was also randomised. All exposure to experimental stimuli and data collection occurred via the online survey platform Qualtrics.

**Figure 1:**
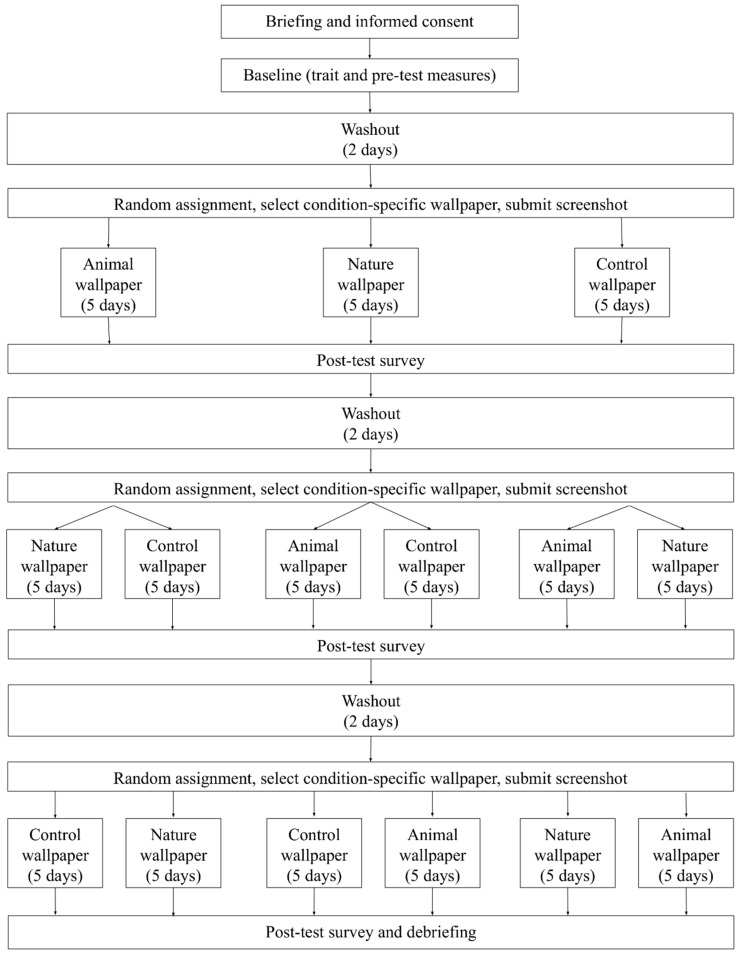
Experimental Flow. *Note.* Participants were randomly assigned to each condition such that each participant completed each condition once. Participants were not aware of the order in which they would go through each condition, nor were they explicitly informed about the difference between each condition.

During the baseline session, participants provided data comprising information on individual differences as well as pretest levels of all outcomes of interest (Table 2). Following that, participants entered three one-week intervention phases (Figure 1). Each phase corresponded to one of three wallpaper conditions (nature, animal, or neutral control), with condition order counterbalanced across participants. Each study phase spanned five days, beginning on Sunday evening and ending on Friday evening. Prior to the start of the first phase and between each phase, a two-day washout period was enforced from Friday night to Sunday night (Figure 1).

At the beginning of each phase (i.e., Sunday night), participants received an email with a link to download 12 wallpapers corresponding to the assigned condition (i.e., nature, animal, or control; see “Experimental Stimuli” section). They selected one wallpaper (from the 12 available wallpapers) to set as their smartphone lock screen and submitted a screenshot to confirm compliance. The chosen wallpaper was used for five consecutive days. At the end of each phase (i.e., Friday night), participants completed a survey assessing posttest confirmatory and exploratory outcomes. Following this survey, participants were instructed to switch to a blank lock screen for the two-day washout period. This procedure was repeated across three consecutive weeks, with participants exposed to a different condition each week.

Upon successful completion of the three-week study, participants filled out a compensation survey, receiving either course credits or cash based on their preference. After data collection concluded, a debriefing email was sent to participants.

#### 2.2.1. Experimental Stimuli

**Figure 2:**
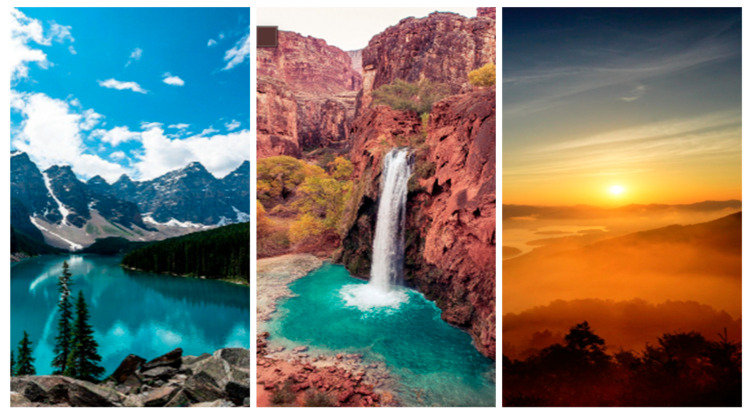

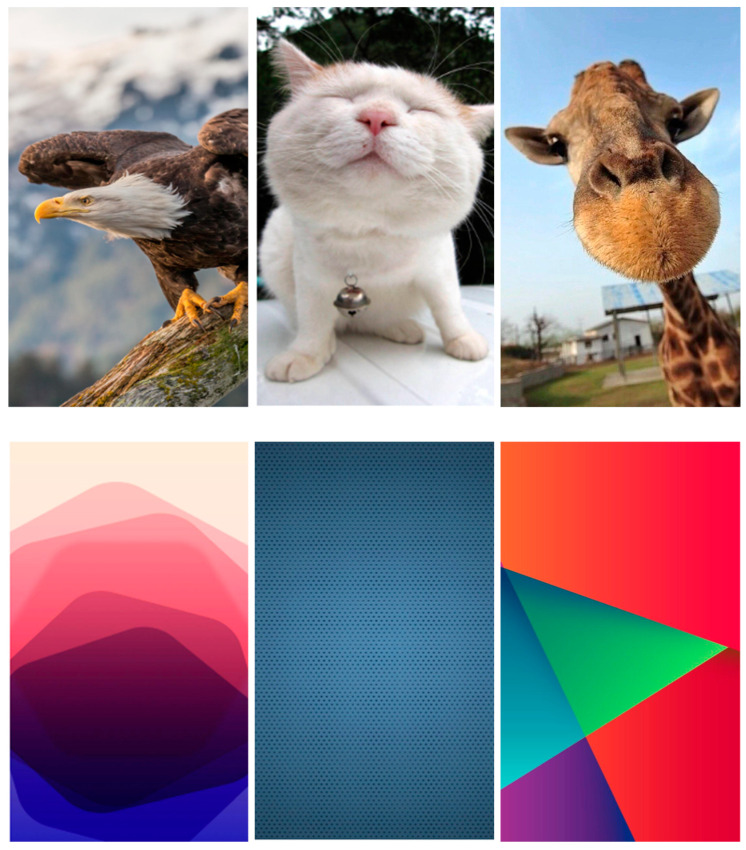
Examples of Lock Screen Wallpapers. *Note.* Examples of nature, animal, and control condition lock screen wallpapers are shown respectively in each row.

Nature, animal, and control lock screen wallpapers were selected to investigate their potential psychological effects on participants, with the selection carried out by the sixth author and approved by the first two authors and the last author. A total of 12 wallpapers were chosen for each condition (nature, animal, and control), for a total of 36 wallpapers (see examples in Figure 2). The wallpapers were selected to evoke specific emotional or neutral responses, following existing literature on environmental imagery, animal interaction, and affect regulation (Gasper et al., 2019; Johnson et al., 2023; Schreuder et al., 2016). Participants were allowed to select from a set of 12 wallpapers for each condition to approximate typical, self-selected lock screen use, facilitating stimulus generalisation (Rescorla & Furrow, 1977; Song et al., 2021).^4^ A description of the wallpapers and the selection process for each condition is provided below.

##### Nature Wallpaper

The nature wallpapers consisted of 12 images of serene natural environments, such as forests, mountains, and bodies of water, specifically chosen for their potential to promote relaxation and reduce stress (see examples in Figure 2). These images were selected to depict nature in isolation, excluding animals and human-made structures to avoid introducing additional variables or distractions.

##### Animal Wallpaper

The animal wallpapers consisted of 12 images featuring both companion animals (e.g., dogs, cats) and non-companion animals (e.g., giraffes, wild birds). Both companion and non-companion animal wallpapers were offered to avoid triggering specific emotional responses (e.g., sadness from recent pet loss) and to ensure a broader assessment of the emotional effects of animal imagery. These wallpapers were chosen for their potential to evoke positive emotional connections commonly associated with animals (see examples in Figure 2).

##### Neutral Wallpaper (Control)

The control wallpapers were used as the control condition and consisted of 12 images designed to evoke minimal emotional response (see examples in Figure 2). These wallpapers lacked a focal point of attraction to ensure that it would not elicit positive or negative emotional reactions from the participants. The selection of wallpapers was curated to provide participants with neutral backgrounds, aimed to minimise any potential influence on participants’ psychological states, as these control wallpapers consisted solely of simple colours, shapes, and lines.

### 2.3. Measures

#### 2.3.1. Individual Differences

All individual differences^5^ were measured during the baseline session.

**Demographics.** Age, sex, and race were obtained during the baseline questionnaire.^6^ Participants self-reported their age at data collection in years, and sex in terms of three options (“*Male*”, “*Female*”, “*Non-Binary*”). Participants were asked to self-report their race from one of four options (“*Chinese*”, “*Malay*”, “*Indian*”, “*Others*”), which corresponds to the primary system for race classification in Singapore (Chua, 2021).

**Dispositional Boredom Proneness.** Each participant’s dispositional boredom proneness was assessed using the 8-item Short Boredom Proneness Scale (Struk et al., 2017; e.g., “I find it hard to entertain myself”, “It takes more stimulation to get me going than most people”) on a 7-point agreement scale (1 = *Highly disagree*, 7 = *Highly agree*). Inter-item consistency was good in the current sample (tau-equivalent reliability, i.e., Cronbach’s α = 0.81, 95% Feldt CI = [0.72, 0.87]).

**Dispositional Smartphone Reliance.** Each participant’s dispositional reliance on their smartphone was assessed using the 10-item Smartphone Addiction Scale–Short Version (Kwon et al., 2013). Participants reported the extent to which a series of statements (e.g., “In general, I miss planned work due to smartphone use”, “In general, I felt pain in the wrists or at the back of the neck while using a smartphone”) applied to them, on a 6-point agreement scale (1 = *Strongly disagree,* 6 = *Strongly agree*). Inter-item consistency was good in the current sample (tau-equivalent reliability, i.e., Cronbach’s α = 0.87, 95% Feldt CI = [0.81, 0.91]).

**Dispositional Lock Screen Engagement.** Participants’ dispositional engagement with their lock screen was assessed with four items developed specifically for the purposes of this study, each tapping on a specific facet of engagement. First, participants indicated how often they *recognised* their smartphone lock screen wallpaper as the specific subject (e.g., a flower or animal) instead of as “the phone lock screen” on a 7-point frequency scale (1 = *Never*, 7 = *Multiple times a week*). Second, participants indicated how often they *change* their lock screen wallpaper on a 7-point frequency scale (1 = *Never*, 7 = *Multiple times a week*). Third, participants indicated how aesthetically *pleasing* they found their current lock screen wallpaper on a 5-point intensity scale (1 = *Not at all*, 5 = *Extremely pleasing*). Fourth, participants indicated how *distracting* they found their current lock screen wallpaper on a 5-point intensity scale (1 = *Not at all*, 5 = *Highly distracting*).

#### 2.3.2. Confirmatory Outcomes

All confirmatory outcomes were assessed as weekly state measures, both during the baseline (pretest) week and at each subsequent posttest week. Where applicable, inter-item consistencies are reflected in Table 3.

**Perceived Stress.** Participants’ pretest and posttest levels of perceived stress were assessed by the question “How stressed did you feel this week overall?” rated on an 11-point scale (0 = *no stress*, 10 = *extreme stress*).

**Negative Affect.** Participants’ pretest and posttest levels of negative affect were assessed with a 9-item measure based on the Circumplex Model of Affect (Russell, 1980). Participants indicated the extent to which they experienced high-arousal negative affect (e.g., angry), medium-arousal negative affect (e.g., nervous), and low-arousal negative affect (e.g., sad). Participants were asked to indicate the extent to which they felt each word for that week on a 5-point scale (1 = *Not at all*, 5 = *Extremely*).

**Positive Affect.** Participants’ pretest and posttest levels of positive affect were assessed with a 9-item measure based on the Circumplex Model of Affect (Russell, 1980). Participants indicated the extent to which they experienced high-arousal positive affect (e.g., energetic), medium-arousal positive affect (e.g., happy), and low-arousal positive affect (e.g., calm). Participants were asked to indicate the extent to which they felt each word for that week on a 5-point scale (1 = *Not at all*, 5 = *Extremely*).

**Life Satisfaction.** Participants’ pretest and posttest levels of life satisfaction were assessed by the question “Taking all things together, how satisfied are you with your life as a whole this week?” rated on a 5-point scale (1 = *Very Dissatisfied*, 5 = *Very Satisfied*).

**Productivity.** Participants’ pretest and posttest levels of productivity were assessed with a 3-item measure (i.e., “This week, I was productive”, “This week, I did a lot of things”, “This week, I did nothing”; Kasturiratna et al., 2025) on a 5-point scale (1 = *Strongly disagree*, 5 = *Strongly agree*).

**Procrastination.** Participants’ pretest and posttest levels of procrastination were assessed with a 5-item measure (e.g., “I put things off so long this week that my well-being or efficiency unnecessarily suffered”, “My week would be better if I did some activities or tasks earlier”; Steel, 2002) on a 5-point scale (1 = *Very seldom or not true to me*, 5 = *Very often or true of me*).

#### 2.3.3. Exploratory Outcomes

Exploratory^7^ outcomes were only collected during the weekly posttest sessions and were not assessed at pretest. Where applicable, inter-item consistencies are reflected in Table 3.

**Lock Screen Engagement.** Participants’ posttest engagement with their smartphone lock screen was assessed using the first, third, and fourth items corresponding to those in the dispositional measure. Specifically, they reported (a) how often they recognised their lock screen wallpaper as the specific subject (e.g., a flower or animal) rather than simply “the lock screen,” on a 5-point scale (1 = *Unsure*, 5 = *Multiple times a day*), (b) how aesthetically pleasing they found their current lock screen wallpaper on a 5-point scale (1 = *Not at all pleasing*, 5 = *Extremely pleasing*), and (c) how distracting they found their current lock screen wallpaper on a 5-point scale (1 = *Not at all distracting*, 5 = *Extremely distracting*).

**Perceived Excessive Smartphone Use.** Participants’ posttest severity of self-perceived excessive smartphone usage was assessed using the 3-item Excessive Internet Use measure (Caplan & High, 2006) on a 6-point scale (1 = *Strongly Disagree*, 6 = *Strongly Agree*). The measure was adapted in the current study to refer to participants’ experiences over the past week by prefacing each item with the phrase “In the past week” before the original items (e.g., “In the past week, I think the amount of time I spent using my smartphone to check for new activity was excessive”).

**Problems with Executive Control.** Participants’ posttest severity of problems with executive control was assessed using a 6-item measure (Buchanan et al., 2010) on a 4-point scale (1 = *No problems experienced*, 4 = *A great many problems experienced*). The measure was adapted in the current study to refer to participants’ experiences over the past week by adding the prompt, “With reference to the past week, please rate the extent to which external factors or circumstances have affected your ability to maintain your attention on a particular task.” Participants then responded to all items (e.g., “Did you find it difficult to keep your attention on a particular task?”, “Did you tend to ‘lose’ your train of thought?”).

**Self-Rated Mental Health.** Participants’ posttest mental health was assessed by the question “In this week, how would you rate your mental health?” rated on a 5-point scale (1 = *Poor*, 5 = *Excellent*).

### 2.4. Analytic Plan

#### 2.4.1. Interpretation

**Frequentist *p*-values.** Statistical significance in the Frequentist framework was determined using traditional *p*-value thresholds (i.e., *p* < 0.050). Where both one-tailed and two-tailed tests were possible, two-tailed tests were used as they are more conservative (i.e., require stronger evidence to reject the null hypothesis).

**Bayes Factors.** For the Bayesian analyses, Bayes Factors (BF_10_) were used to compare the alternative model (i.e., with the variable of interest) against a null model, providing an estimate of the strength of evidence for the alternative hypothesis. BF_10_s were interpreted following M. D. Lee and Wagenmakers (2013). Specifically, BF_10_ < 0.333 would indicate at least moderate evidence for the null hypothesis, 0.333 ≤ BF_10_ < 1.000 would indicate only anecdotal evidence for the null hypothesis, BF_10_ = 1.000 would indicate no evidence for either hypothesis, 1.000 < BF_10_ ≤ 3.000 would indicate only anecdotal evidence for the alternative hypothesis, and 3.000 < BF_10_ would indicate at least moderate evidence for the alternative hypothesis.

**Magnitude of effects.** The magnitude of all effects in the form of η^2^*_g_* were interpreted following Bakeman (2005). Specifically, 0.020 ≤ η^2^*_g_* < 0.130 would indicate a small effect, 0.130 ≤ η^2^*_g_* < 0.260 would indicate a medium effect, and 0.260 ≤ η^2^*_g_* would indicate a large effect.

#### 2.4.2. Pre-Registered Analyses

The current study included six key dependent variables as per the pre-registration: stress, negative affect, life satisfaction, positive affect, productivity, and procrastination. As outlined in the pre-registration, for each dependent variable, we planned to conduct both a two-way 3 (within-subject condition: nature vs. animal vs. control) × 6 (between-subject order of conditions) mixed ANOVA and a one-way repeated measures ANOVA (within-subject condition: nature vs. animal vs. control). However, we deviated slightly from the pre-registered analysis plan for methodological reasons. Specifically, the one-way repeated measures ANOVA was conducted only when the two-way mixed ANOVA revealed no significant interaction between condition and order. This decision was based on the principle that a significant interaction implies that the effect of condition varies as a function of presentation order; under such circumstances, collapsing across order would obscure these differences and yield estimates that are difficult to interpret. Accordingly, restricting the one-way analyses to cases without a significant interaction ensured that the reported condition effects were not conflated with order-dependent variation.

As pre-registered, both Frequentist and Bayesian approaches were employed. For the Bayesian analyses, distinct null models were specified for the two analyses. For the two-way mixed ANOVA, the Bayesian null model included only the main effect of condition and excluded both the order main effect and the condition × order interaction, providing a baseline for assessing the cumulative contribution of order-related terms. For the one-way repeated-measures ANOVA, the Bayesian null model consisted solely of an intercept term (i.e., no predictors), serving as a baseline against which the effect of condition was evaluated.

Post hoc pairwise comparisons were conducted only when both (1) Frequentist statistical significance was reached (*p* < 0.050) and (2) the Bayes Factor indicated at least moderate evidence for an effect (i.e., BF_10_ > 3.000). The *p*-values obtained from Frequentist post hoc pairwise comparisons were adjusted using Bonferroni correction to control for familywise error. Bayes Factors (BF_10_) were computed for each pairwise contrast to assess the relative strength of evidence for differences between conditions. These decision criteria for conducting post hoc comparisons were not specified in the pre-registration but were introduced to ensure greater consistency and evidential robustness across analytic frameworks.

#### 2.4.3. Exploratory Analyses

**Additional Outcomes.** Three outcomes related to lock screen engagement (i.e., recognition frequency, visual appeal, and distraction) and three additional outcomes serving as more distal proxies of well-being (i.e., problems with executive control, excessive smartphone use, mental health) were analysed as additional dependent variables. Analyses of the six additional outcomes were treated as one set and had their *p*-values corrected for multiple comparisons using the Hommel procedure (Vickerstaff et al., 2019).

**Moderation by Individual Differences.** To explore whether individual differences moderated the effects of the lock screen intervention, we tested interactions between condition and two separate dispositional variables: boredom proneness and smartphone reliance. For each dependent variable (both the original confirmatory outcomes and the additional exploratory outcomes), we fit a linear mixed-effects model including condition, the moderator, and their interaction, with a random intercept for participants. Omnibus tests of fixed effects were obtained using Type III sum of squares. Analyses of the 12 outcomes across both moderators (i.e., 24 analyses in total) were treated as one set and had their *p*-values corrected for multiple comparisons using the Hommel procedure (Vickerstaff et al., 2019). If the condition × dispositional interaction reached significance (*p*_Hommel_ < 0.050), we probed the effect using simple slopes analyses and visualised the interaction with predicted values and confidence intervals.

## 3. Results

### 3.1. Pre-Registered Confirmatory Outcomes

**Figure 3:**
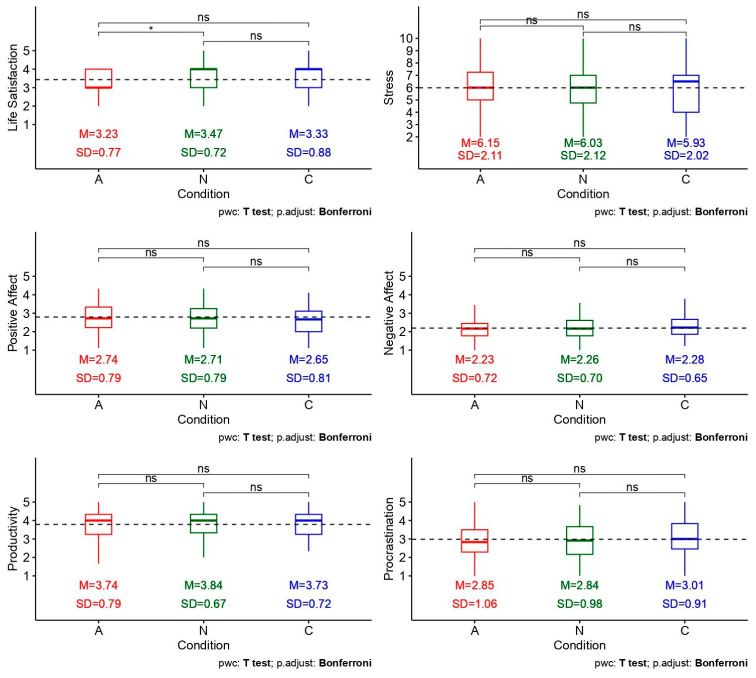
Patterns of Responses Across Conditions for the Six Confirmatory Outcomes. *Note. N* = 60. The main effect of condition was non-significant for all outcomes. Pretest levels are indicated by the dotted horizontal line. * indicates statistically significant pairwise differences (with the number of asterisks corresponding to the smallness of the *p*-value) while ns indicates non-significant pairwise differences.

No evidence was found for order effects in the two-way mixed ANOVAs on any of the six confirmatory outcomes (stress, negative affect, positive affect, life satisfaction, productivity, procrastination), with all condition × order interaction *p*s ≥ 0.298 from Frequentist analyses. Bayesian analyses suggested support for the null model, with all BF_10_s = [0.018, 0.215], indicating that the alternative model was only 0.018 to 0.215 times more likely to be true than the null model (or, conversely, the null model was 4.651 to 55.556 times more likely to be true than the alternative model). Thus, all outcomes were analysed with order collapsed (Table 4 and Figure 3); no evidence was found for the main effect of condition on any of the six confirmatory outcomes, with all condition *p*s ≥ 0.098 and BF_10_s = [0.066, 0.433] (i.e., the null model was 2.309 to 15.151 times more likely to be true than the alternative model). Categorically, Bayesian evidence for the null model ranged from anecdotal in magnitude (for life satisfaction) to strong in magnitude (for stress, negative affect, and positive affect).

### 3.2. Non-Registered Exploratory Outcomes

**Figure 4:**
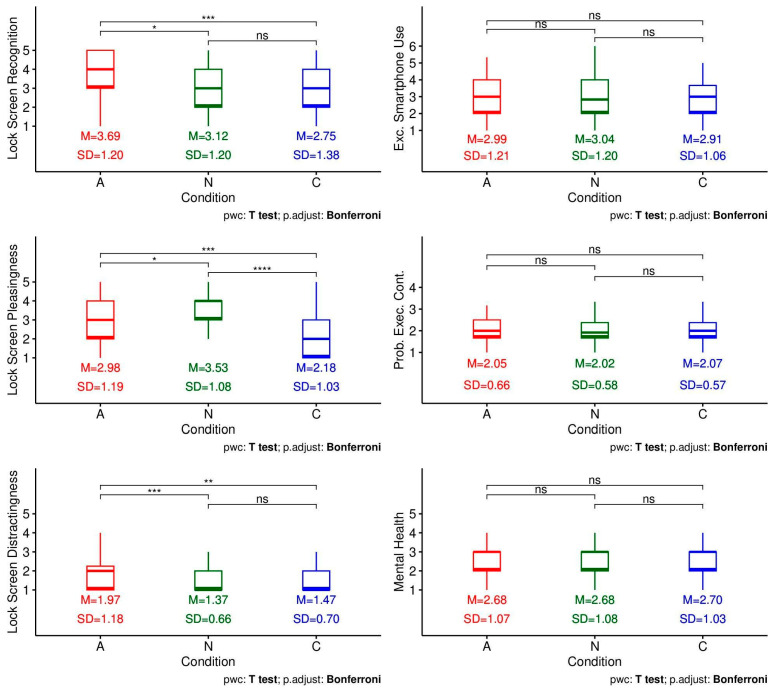
Patterns of Responses Across Conditions for the Six Exploratory Outcomes. *Note. N* = 60 for all outcomes except lock screen recognition with *N* = 52. The main effect of condition was significant for the lock screen-related outcomes, and non-significant for all other outcomes. Pretest levels were not measured for exploratory outcomes. *, **, ***, **** indicate statistically significant pairwise differences (with the number of asterisks corresponding to the smallness of the *p*-value) while ns indicates non-significant pairwise differences.

No evidence was found for order effects in the two-way mixed ANOVAs on any of the six exploratory outcomes (lock screen recognition, lock screen pleasingness, lock screen distractingness, excessive smartphone use, problems with executive control, mental health), with all condition × order interaction *p*_Hommel_s ≥ 0.096. Thus, all outcomes were analysed with order collapsed (Table 4 and Figure 4); no evidence was found for the main effect of condition on three of the exploratory outcomes (excessive smartphone use, problems with executive control, mental health), with condition *p*_Hommel_s ≥ 0.986.

However, the main effect of condition was observed to a small extent on lock screen recognition (*F*(2, 102) = 11.53, *p*_Hommel_ < 0.001, η^2^*_g_* = 0.09) and lock screen distractingness (*F*(1.40, 82.34) = 12.85, *p*_Hommel_ < 0.001, η^2^*_g_* = 0.08), and to a medium extent on lock screen pleasingness (*F*(2, 118) = 23.98, *p*_Hommel_ < 0.001, η^2^*_g_* = 0.20). Upon probing, similar condition-wise patterns were observed for recognition and distractingness: there was no evidence of a significant difference between the nature and control conditions, but there were significantly higher levels of recognition and distractingness in the animal condition versus the control condition, and similarly in the animal condition versus the nature condition. On the other hand, a different pattern was observed for pleasingness, where the nature condition caused significantly higher levels of pleasingness than both the animal and control conditions, and the animal condition in turn caused significantly higher levels of pleasingness as compared to the control condition.

### 3.3. Non-Registered Exploratory Moderators

No evidence was found for the moderating role of either dispositional variable (i.e., dispositional boredom proneness, dispositional smartphone reliance) on any of the confirmatory nor exploratory outcomes, with all condition × dispositional moderator interaction terms having *p*_Hommel_s ≥ 0.089.

## 4. Discussion

The current 3-week within-subject randomised cross-over trial examined whether brief exposure to static nature- and animal-themed smartphone lock screen wallpapers could improve everyday life outcomes in contexts where direct access to nature and animals is limited. To address this aim, the study assessed both primary psychological indicators (i.e., perceived stress, negative affect, positive affect, and life satisfaction) as well as secondary behavioural indicators (i.e., productivity and procrastination) arising from exposure to these wallpapers. In addition, we explored the impact of the lock screen wallpapers on more proximal exploratory outcomes, namely lock screen recognition, pleasingness, and distractingness. A total of 60 participants each completed three one-week intervention phases, during which they were exposed to nature, animal, and neutral control wallpapers in a counterbalanced order. The wallpapers were standard lock screen images, presented in a typical, everyday format without any audio or additional multisensory features.

Across the six pre-registered confirmatory outcomes (i.e., stress, negative affect, positive affect, life satisfaction, productivity, and procrastination), Frequentist analyses failed to show evidence of any differences in outcomes attributable to condition. Bayesian analyses gave further evidence for null effects, with BF_10_s ranging from 0.066 to 0.433, indicating it was approximately 2 times to 15 times more likely that there was no effect at all (vs. some effect). On the other hand, analyses of exploratory outcomes revealed nuanced patterns: animal-themed wallpapers were associated with greater recognition (η^2^*_g_* = 0.09) and distraction (η^2^*_g_* = 0.08) than nature-themed or neutral control wallpapers to a small extent, while nature-themed wallpapers were evaluated as more aesthetically pleasing (η^2^*_g_* = 0.20) than both animal and control images to a medium extent. These attentional and hedonic differences, however, did not translate into broader well-being or behavioural benefits. Additionally, across both confirmatory and exploratory outcomes, dispositional traits did not moderate any effects of condition. The potential reasons behind these non-significant findings, and their implications, are elucidated in the following sections.

### 4.1. Brief Digital Exposure

Across all pre-registered confirmatory outcomes, no meaningful differences emerged between conditions, indicating that brief, passive exposure to static nature- or animal-based lock screen wallpapers over the brief intervention periods examined in the current work was insufficient to produce robust changes in stress and everyday life outcomes. Importantly, these findings should be interpreted with reference to the current specific form of incidental exposure, in which participants encountered the images passively, rather than as evidence about brief digital exposure more broadly. It remains possible that more active forms of engagement with nature- or animal-based digital content, such as longer viewing durations, interactive formats, or more immersive experiences may produce different effects.

Our findings are consistent with previous works, where immersive yet short-lived forms of digital nature exposure, such as brief nature videos, do not reliably restore attentional resources or broader cognitive functioning despite strong theoretical motivation (e.g., Hartanto et al., 2023; Hicks et al., 2020). More broadly, the findings also align with evidence that minimal or incidental interventions exert limited influence on well-being unless they involve sustained engagement (Bell et al., 2025; Browning et al., 2020a; Guevarra et al., 2025; Kahn et al., 2008; Kimura et al., 2021).

According to Attention Restoration Theory, restorative environments are characterised by being away, fascination, extent, and compatibility (S. Kaplan, 1995; R. Kaplan & Kaplan, 1989). In the current lock screen wallpaper paradigm, each of these properties was likely reduced. Viewing a wallpaper on one’s smartphone may afford a minimal sense of being away, as the image appears within the same device that is tied to daily obligations and ongoing demands. Furthermore, because the images were static and repeatedly encountered, they may evoke only weak or momentary fascination. Likewise, a single wallpaper offers limited extent, lacking the richness and immersiveness of a broader environment. Compatibility may also be constrained, as exposure to the wallpaper occurs during routine smartphone use rather than doing so out of personal preference or intrinsic motivation. Accordingly, fleeting visual encounters are unlikely to provide the depth or duration required to restore directed attention. The present findings therefore suggest that the restorative potential of nature- and animal-based visual cues depends less on content per se than on whether the mode of delivery can generate the core restorative properties proposed by the Attention Restoration Theory.

Exploratory analyses revealed that wallpaper categories produced reliably different and largely trait-invariant responses. Animal-themed wallpapers were associated with greater self-reported levels of recognition and distraction, whereas nature-themed wallpapers were rated as more pleasing than animal or control images. Although these findings should be interpreted cautiously given their exploratory nature, the dissociation between attentional salience and hedonic appraisal may represent a theoretically informative contribution. Specifically, this pattern is consistent with the possibility that animal stimuli were more effective at triggering bottom-up attentional capture, consistent with accounts proposing that animals automatically capture attention due to their relevance for survival (Hu et al., 2023; Yang et al., 2024). However, heightened attentional engagement did not translate into broader well-being benefits. This suggests that noticing a stimulus more, or finding it distracting, may be functionally distinct from engaging with it in a way that supports restoration or affective improvement. In this vein, the present findings offer a useful starting point for future research by examining how visual stimuli may differentially influence attentional salience and how they contribute to downstream consequences.

Additionally, dispositional traits of boredom proneness and smartphone reliance did not moderate the effects of lock screen wallpapers on either the confirmatory or exploratory outcomes, indicating that responses to lock screen imagery were relatively uniform across individuals. In the broader behavioural change literature, traits such as boredom proneness and self-control have been linked to disengagement from nudge-based mobile interventions (e.g., just-in-time intervention; J. Park & Lee, 2023). Against this backdrop, the absence of moderation in the present study is more consistent with relatively automatic, stimulus-driven responses to lock screen imagery than with reflective, trait-dependent processes typically implicated when individual differences drive sustained behavioural or well-being change (Bargh & Chartrand, 1999; De Ridder et al., 2022; Kahneman, 2011).

### 4.2. Implications

The present findings help clarify whether exposure to nature- or animal-based elements can replicate previously observed benefits for affect and related outcomes. Prior daily diary research suggests that individuals report higher positive affect on days with greater exposure to natural environments or animals (e.g., Goh et al., 2023). However, the present study did not replicate these benefits. Across both the nature and animal wallpaper conditions, we found no evidence of improvements in positive affect nor in the other three primary psychological outcomes, (i.e., life satisfaction, stress, and negative affect). This divergence from prior work suggests that the effects observed in naturalistic contexts may not generalise to brief, incidental digital exposures.

A similar pattern emerged for behavioural outcomes. We did not find support for improvements in procrastination or productivity following exposure to either nature or animal wallpapers. Taken together, the present findings indicate that low-intensity, passive exposure may be insufficient to translate into measurable changes in everyday behaviour. This is consistent with prior work suggesting that even relatively immersive but short digital exposures yield limited benefits unless engagement is more sustained or effortful (Aguilar et al., 2025; Hartanto et al., 2023; Kahn et al., 2008; Schertz & Berman, 2019).

More broadly, the results qualify expectations from the Biophilia Hypothesis. Although humans have an innate affinity for nature and living organisms (Wilson, 1984), our null findings indicate that any biophilic response elicited by the lock screen wallpapers was too weak to produce tangible improvements in well-being or behaviour. It may be possible that biophilic effects require more salient, multi-sensory engagement rather than passive, single-modality exposure to static digital imagery (Browning et al., 2020b; Cai et al., 2025; Franco et al., 2017; Gaekwad et al., 2022; Kim et al., 2024; Lin et al., 2025; Ulrich, 1983). As a whole, the results point to the importance of engagement intensity and modality. The benefits reported in immersive or real-world contexts may depend less on content alone and more on the depth and quality of interaction (Berto, 2014; Capaldi et al., 2014; Ulrich et al., 1991; White et al., 2019), which were intentionally minimal in the present paradigm. While our study did not show immediate benefits from exposure to nature or animal lock screen wallpapers, it serves as an important step in understanding the nuances of passive digital interventions and their role in well-being. As digital tools continue to be incorporated into daily life, understanding their limitations and potential is essential for developing effective interventions.

### 4.3. Strengths of the Current Study

This study has several methodological strengths that enhance the robustness and interpretability of its findings. First, it prioritised real-world smartphone use over highly controlled laboratory exposure, with participants engaging with the lock screen wallpapers in their everyday environments. Additionally, exposure duration was not experimentally fixed but was based on the participants’ personal engagement time with their lock screens. This also meant that participants could repeatedly view these images as often as they would naturally view their lock screens, as part of their individual phone-checking behaviour. No additional constraints were added on the viewing environment of these wallpapers. This design enhances ecological validity by integrating the intervention within participants’ real-world usage patterns, and supports the generalisability of the results to naturalistic smartphone behaviour (Faria et al., 2023; Keidser et al., 2020). Second, the inclusion of a neutral control condition enabled the effects of lock screen imagery to be disentangled from general smartphone exposure or novelty effects, thereby strengthening causal interpretation and reducing alternative explanations for the observed effects (Boot et al., 2013; Boutron et al., 2007). Third, the use of a randomised cross-over design with counterbalancing ensured that all participants experienced every condition, thereby minimising the influence of stable individual differences (Charness et al., 2012; Senn, 2002) and potential order effects (J. L. Brooks, 2012). Consistent with this rationale, analyses explicitly modelling order revealed no evidence of order effects. Potential carryover effects were further mitigated through the inclusion of a two-day washout period between conditions.

In addition, several measurement and analytic features further strengthen the rigour of the current work. First, as the confirmatory outcomes were also measured at pretest levels during the baseline session, observed changes could be interpreted relative to participants’ own initial levels rather than solely as differences between conditions, thereby clarifying whether each condition was associated with improvements or deteriorations relative to baseline. Second, strong internal consistency across measures further supports the reliability of the instruments employed, and hence the overall findings (Cronbach, 1951; McNeish, 2018). Third, the use of both Frequentist and Bayesian analytic frameworks provided complementary evidence regarding the presence and strength of effects, contributing to more robust and nuanced inference (Kruschke & Liddell, 2018; Wagenmakers et al., 2018). Finally, pre-registration of the study design and analytic plan reduced researcher degrees of freedom, enhancing the credibility and transparency of the findings (Nosek et al., 2018).

Notably, although personality factors were not directly measured, aspects of the study design and analyses mitigate this concern. The within-subject design controlled for stable individual differences, such as personality factors, by treating each participant as their own control, thereby holding between-person variability constant when estimating the effects of the wallpaper manipulation. Moreover, moderation analyses showed that dispositional boredom proneness and dispositional smartphone reliance did not influence the within-subject effects of the intervention, as interaction effects were consistently non-significant. Collectively, these indicate that unmeasured personality traits are unlikely to drive the observed effects.

### 4.4. Limitations and Future Directions

A key limitation concerns the intensity of the intervention. As previously hinted at, overly simplistic interventions such as changing one’s lock screen wallpaper may lack the immersion required for affective or stress-regulatory changes to emerge. This is consistent with work showing that while even very short exposures can improve positive affect (e.g., Goh et al., 2023), more sustained interventions are often needed for reducing stress levels (Heber et al., 2017) and inducing behavioural change (Breitwieser et al., 2020). Future studies could therefore consider incorporating occasional active prompts to help convert automatic attentional responses into more reflective regulation (Fogg, 2009; Nahum-Shani et al., 2018).

An additional consideration is the heterogeneity introduced by allowing participants to select their preferred wallpaper within each condition. While this approach enhances ecological validity by reflecting naturalistic device use, it may also introduce variability in visual exposure that could attenuate observed effects. Exploratory analyses suggested that variance attributable to specific wallpaper choice was minimal for most outcomes, though small non-zero effects were observed for stress and procrastination. Although these findings are preliminary, they raise the possibility that certain image-level characteristics may differentially influence outcomes. Future research may benefit from systematically manipulating or controlling such characteristics to better isolate the active components of digital nature and animal exposure.

Additionally, the generalisability of the findings is limited by the characteristics of the sample. Participants were Generation Z university students from a single institution in Singapore, a highly urbanised context with limited access to natural environments. Cultural context may shape how individuals perceive and respond to nature- and animal-related stimuli—for example, through differences in familiarity, aesthetic preferences, or the symbolic meanings attached to such content. The educational context may also matter, as university students tend to have relatively homogeneous routines, cognitive demands, and patterns of device use, which could influence both exposure to and engagement with lock screen stimuli. As such, the results may not readily generalise beyond this specific age group, cultural and educational context, or environmental setting.

Additionally, the participants had high levels of digital engagement due to the ubiquity of smartphones in the Singapore population (Statista, 2024), which may constrain generalisability to populations with lower levels of digital engagement. Hence, replication across contexts within and beyond Singapore, as well as in populations outside of urban, young, and heavy smartphone users would help establish the robustness of these findings. This is particularly important given that responses to passive visual cues may differ systematically due to variations in age, neurocognitive profile, and psychological vulnerability. Examining such groups will help determine whether passive visual interventions have broader applicability or whether their effects remain context- and population-specific.

## 5. Conclusions

The present study shows that brief, passive exposure to nature- and animal-themed smartphone lock screen wallpapers does not robustly improve levels of stress and everyday life outcomes. While such imagery can shape momentary attention and aesthetic experience, the effects were short-lived and did not extend to broader well-being and behavioural changes. Animal images tended to heighten recognition and distraction, whereas nature images were viewed more positively, highlighting a distinction between what captures attention and what feels pleasant. The findings help clarify when digital nature or animal exposure may be helpful and when its influence is likely to remain limited. Future work should therefore examine longer interventions, diverse populations, and designs that pair visual cues with more active or sustained self-regulatory support.

## Figures and Tables

**Table 1:** Snapshot of Existing Studies on Benefits of Digital Nature/Animal Interventions.

Study	Duration of Exposure	Outcome
Exposure via Video		
Grassini et al. (2022)	3 min/video × 4 videos	Increase in perceived relaxation
Hartanto et al. (2023)	6 min	No significant change in affect
Exposure via VR		
Anderson et al. (2017)	15 min/scene × 3 scenes	Decrease in negative affect
Browning et al. (2020a)	6 min	Decrease in negative affect
Chan et al. (2023)	5 min	Increase in positive affect
Leung et al. (2022)	6–10 min/session × 2–3 sessions	Decrease in negative affect

*Note*. VR = virtual reality.

**Table 2:** Descriptive Statistics.

Variable	*N*	*M* or %	*SD*	Observed Range	Theoretical Range
**Individual differences**					
***Year of Participation in Study***					
2023	10	17%			
2024	47	78%			
2025	3	5%			
***Demographics***					
Age at data collection (years)	60	21.57	1.59	18–26	18–26
Ethnicity (% Chinese)	60	83%			
Sex (%)					
Female	45	75%			
Male	15	25%			
Non-binary	0	0%			
***Dispositional tendencies***					
Boredom proneness	60	3.33	1.04	1.00–5.88	1.00–7.00
Smartphone reliance	60	3.00	0.98	1.00–5.20	1.00–6.00
***Dispositional lock screen engagement***					
Recognition frequency ^1^	58	5.16	1.59	1.00–7.00	1.00–7.00
Wallpaper change frequency ^1^	59	2.12	0.72	1.00–6.00	1.00–7.00
Visual appeal	60	3.95	1.00	1.00–5.00	1.00–5.00
Distraction	60	1.27	0.61	1.00–4.00	1.00–5.00
**Weekly objective smartphone use (hours) ^2^**					
Pretest	60	20.78	15.36	1–70	0–168
Posttest, nature condition	60	27.59	16.90	2–60	0–168
Posttest, animal condition	60	25.11	15.75	2–54	0–168
Posttest, control condition	60	29.45	15.26	3–56	0–168
**Pretest levels of confirmatory outcomes**					
***Well-being***					
Perceived stress	60	5.98	2.33	0–10	0–10
Negative affect	60	2.19	0.82	1.00–5.00	1.00–5.00
Positive affect	60	2.79	0.79	1.00–4.22	1.00–5.00
Life satisfaction	60	3.43	0.93	1.00–5.00	1.00–5.00
***Behaviour***					
Productivity	60	3.79	0.85	1.00–5.00	1.00–5.00
Procrastination	60	2.98	1.10	1.00–5.00	1.00–5.00

*Note.* ^1^ Reduced *N* reflects the inclusion of an ‘Unsure’ response option for these items. ^2^ Objective smartphone use was verified using participant-submitted screenshots. Further information is available in the current work’s associated ResearchBox.

**Table 3:** Internal Consistency of Weekly Measures.

Measure	Pretest	Nature	Animal	Control
Confirmatory outcomes				
Negative affect	0.92 [0.90–0.93]	0.87 [0.81–0.91]	0.88 [0.82–0.92]	0.85 [0.79–0.90]
Positive affect	0.93 [0.92–0.95]	0.93 [0.90–0.96]	0.93 [0.90–0.95]	0.94 [0.92–0.96]
Productivity	0.80 [0.74–0.84]	0.72 [0.57–0.82]	0.79 [0.67–0.87]	0.76 [0.62–0.85]
Procrastination	0.94 [0.92–0.95]	0.91 [0.88–0.94]	0.94 [0.91–0.96]	0.90 [0.86–0.94]
Exploratory outcomes				
Excessive smartphone use		0.91 [0.86–0.94]	0.94 [0.91–0.96]	0.94 [0.91–0.96]
Problems with executive control		0.84 [0.77–0.89]	0.90 [0.85–0.93]	0.84 [0.76–0.89]

*Note*. *N*_participants_ = 60, *N*_observations_ = 180. Values refer to tau-equivalent inter-item consistency (i.e., Cronbach’s α) and corresponding 95% CI. Inter-item consistency is not applicable for single-item outcomes (i.e., life satisfaction and perceived stress).

**Table 4:** Main Effect of Experimental Condition on Confirmatory and Exploratory Outcomes.

Outcomes	*N*	*F*	*df*_1_	*df*_2_	*p*	BF_10_	η^2^_g_
Confirmatory Outcomes							
Stress	60	0.31	2.00	118.00	0.737	0.075	0.00
Negative Affect	60	0.17	2.00	118.00	0.841	0.066	0.00
Positive Affect	60	0.46	2.00	118.00	0.631	0.093	0.00
Life Satisfaction	60	2.37	2.00	118.00	0.098	0.433	0.02
Productivity	60	0.85	2.00	118.00	0.432	0.116	0.01
Procrastination	60	1.10	1.81	107.02	0.333	0.143	0.01
Exploratory Outcomes							
Lock Screen Recognition ^1^	52	11.53	2.00	102.00	<0.001	-	0.09
Lock Screen Pleasingness	60	23.98	2.00	118.00	<0.001	-	0.20
Lock Screen Distractingness	60	12.85	1.40	82.34	<0.001	-	0.08
Excessive Smartphone Use	60	0.47	2.00	118.00	0.986	-	0.01
Problems with Executive Control	60	0.30	2.00	118.00	0.986	-	0.01
Mental Health	60	0.01	2.00	118.00	0.986	-	0.00

*Note.* ^1^ Reduced *N* due to the inclusion of the “Unsure” response option in the responses for lock screen recognition. Confirmatory outcomes are reported with raw *p*-values, whereas exploratory outcomes are reported with Hommel-corrected *p*-values.

## Data Availability

The current work’s design and its analysis plan were pre-registered. In our pre-registration, we initially included mobile technology engagement as one of the constructs to be measured. However, prior to data collection, the measurement of this construct was excluded due to concerns about the unclear psychometric properties of the scale. Consequently, no data was collected for this construct in the current study. The pre-registration, all materials (including Qualtrics QSF files), data, and analytic script will be made publicly available on ResearchBox (#3072; https://researchbox.org/3072; accessed on 14 April 2026) upon publication. With the exception of Bayesian analyses, all statistical procedures were conducted in R version 4.5.0 (R Core Team, 2021) using the following packages: *dplyr* version 1.2.0 (Wickham et al., 2014), *tidyr* version 1.3.1 (Wickham et al., 2025), *psych* version 2.5.6 (Revelle, 2007), *rstatix* version 0.7.3 (with automatic corrections when assumptions such as sphericity are violated, which can result in non-integer or otherwise atypical degrees of freedom for *F*-tests; Kassambara, 2019), *lmerTest* version 3.1-3 (Kuznetsova et al., 2017), *ggplot2* version 3.5.2 (Wickham, 2016), and *ggpubr* version 0.6.1 (Kassambara, 2016). Bayesian analyses were conducted in JASP version 0.16.2 (with default settings of *r* scale = 0.5 for fixed effects, 1 for random effects, 0.354 for covariates, and uniform model prior; JASP Team, 2023).

## Notes

1 This effect size was obtained by setting the within-subject correlation to 0.50, with *M*animal = 1, *M*nature = 1, *M*control = 0, and *SD*common = 2.844. This effect size could also be obtained by setting the within-subject correlation to 0.25 with the same means and *SD*common = 2.322, or by setting the within-subject correlation to 0.75 with the same means and *SD*common = 4.022. Readers are encouraged to refer to Lakens and Caldwell’s (2021) work for further explanation.

2 As agreed upon between the participants and the research team prior to the start of data collection, any data which was not complete would be deleted. Thus, existing partial data from the 24 individuals who encountered the technical error and nine individuals who did not provide complete responses was deleted, and thus was not available for analysis.

3 No evidence was found for year effects in two-way mixed ANOVAs on any of the six confirmatory outcomes (stress, negative affect, positive affect, life satisfaction, productivity, procrastination) nor on any of the six exploratory outcomes (lock screen recognition, lock screen pleasingness, lock screen distractingness, excessive smartphone use, problems with executive control, mental health), with all condition × year interaction *p*s ≥ 0.132 (confirmatory outcomes) and *p*Hommels ≥ 0.282 (exploratory outcomes) from Frequentist analyses. We thank an anonymous reviewer for highlighting this concern.

4 This design increases ecological validity but introduces heterogeneity in visual exposure. To assess the potential impact of this variability, we conducted exploratory analyses on a random subset of 30 participants, coding the specific images selected and estimating variance in the levels of the six confirmatory outcomes (i.e., stress, negative affect, positive affect, life satisfaction, procrastination, and productivity) attributable to wallpaper choice (nested within condition) after partitioning out variance attributable to condition itself and to individual differences. Non-zero variance attributable to wallpaper choice was observed only for stress (variance = 0.458) and procrastination (variance = 0.015), with virtually zero variance for the remaining outcomes. Details are available in the current work’s associated ResearchBox and should be interpreted cautiously given the exploratory nature and reduced sample.

5 Dispositional levels of problems with executive control and excessive smartphone use were also measured for exploratory purposes but were ultimately not used in the current work. Data on these measures remains available in the main dataset in the current work’s ResearchBox.

6 Academic performance, operationalised as participants’ Grade Point Average scores, was also intended to be collected. However, due to a technical error during the data collection procedure, Grade Point Average scores were incomplete and were thus not used in the current work.

7 Exposure to minor stressors and to minor uplifts, as well as self-perceived levels of busyness, were also measured for exploratory purposes but were ultimately not used in the current work. Data on these measures remains available in the main dataset in the current work’s ResearchBox.
